# Supplementary material for: Comparative single-cell transcriptomic atlases of drosophilid brains suggest glial evolution during ecological adaptation
Source: PLoS Biol. 2025 Apr 29;23(4):e3003120. doi: 10.1371/journal.pbio.3003120 (PMC12040179; doi:10.1371/journal.pbio.3003120)
Supplement: S4 Fig — (A) Glial cell types. Left, UMAP plots of the glial cell type annotation for integrated and disintegrated single-cell transcriptomic atlases of D. melanogaster, D. simulans, and D. sechellia. Right, expression levels of marker genes distinguishing the glial cell types. (B) Kenyon cell types. Left, UMAP plots of the Kenyon cell type annotation for integrated and disintegrated single-cell transcriptomic atlases of D. melanogaster, D. simulans, and D. sechellia. Right, expression levels of marker genes distinguishing the Kenyon cell types. (C) Monoaminergic cell types. Left, UMAP plots of the monoaminergic cell type annotation for integrated and disintegrated single-cell transcriptomic atlases of D. melanogaster, D. simulans, and D. sechellia. Right, expression levels of marker genes distinguishing the monoaminergic cell types. (D) Clock cell types. Left, UMAP plots of the Clock cell type annotation for integrated and disintegrated single-cell transcriptomic atlases of D. melanogaster, D. simulans, and D. sechellia. Right, expression levels of marker genes distinguishing the Clock cell types. (E) Poxn cell types. Left, UMAP plots of the Poxn cell type annotation for integrated and disintegrated single-cell transcriptomic atlases of D. melanogaster, D. simulans, and D. sechellia. Right, expression levels of marker genes distinguishing the Poxn cell types. (F) fru cell types. Left, UMAP plots of the fru cell type annotation for integrated and disintegrated single-cell transcriptomic atlases of D. melanogaster, D. simulans, and D. sechellia. Right, expression levels of marker genes distinguishing the fru cell types. (G) Neuropeptidergic cell types. Left, UMAP plots of the neuropeptidergic cell type annotation for integrated and disintegrated single-cell transcriptomic atlases of D. melanogaster, D. simulans, and D. sechellia. Right, expression levels of marker genes distinguishing the neuropeptidergic cell types. (H) Cholinergic cell types. Left, UMAP plots of the choline [file pbio.3003120.s010.pdf]

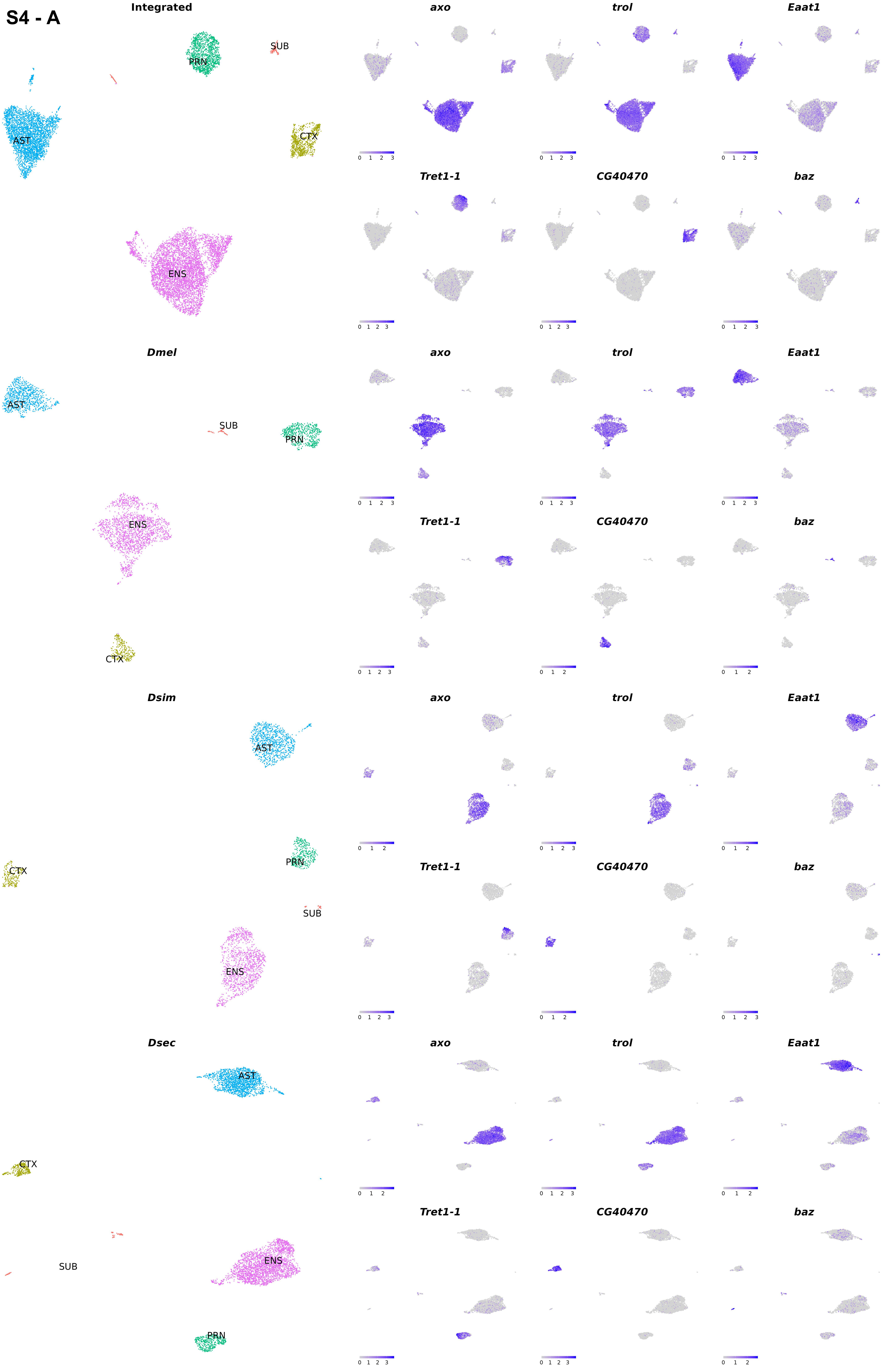

S4 - A
Integrated
axo
trol
Eaat1
SUB
PRN
AST
CTX
Tret1-1
CG40470
baz
ENS
Dmel
axo
trol
Eaat1
AST
SUB
PRN
Tret1-1
CG40470
baz
ENS
CTX
Dsim
axo
trol
Eaat1
AST
Tret1-1
CG40470
baz
CTX
PRN
SUB
ENS
Dsec
axo
trol
Eaat1
AST
CTX
Tret1-1
CG40470
baz
SUB
ENS
PRN

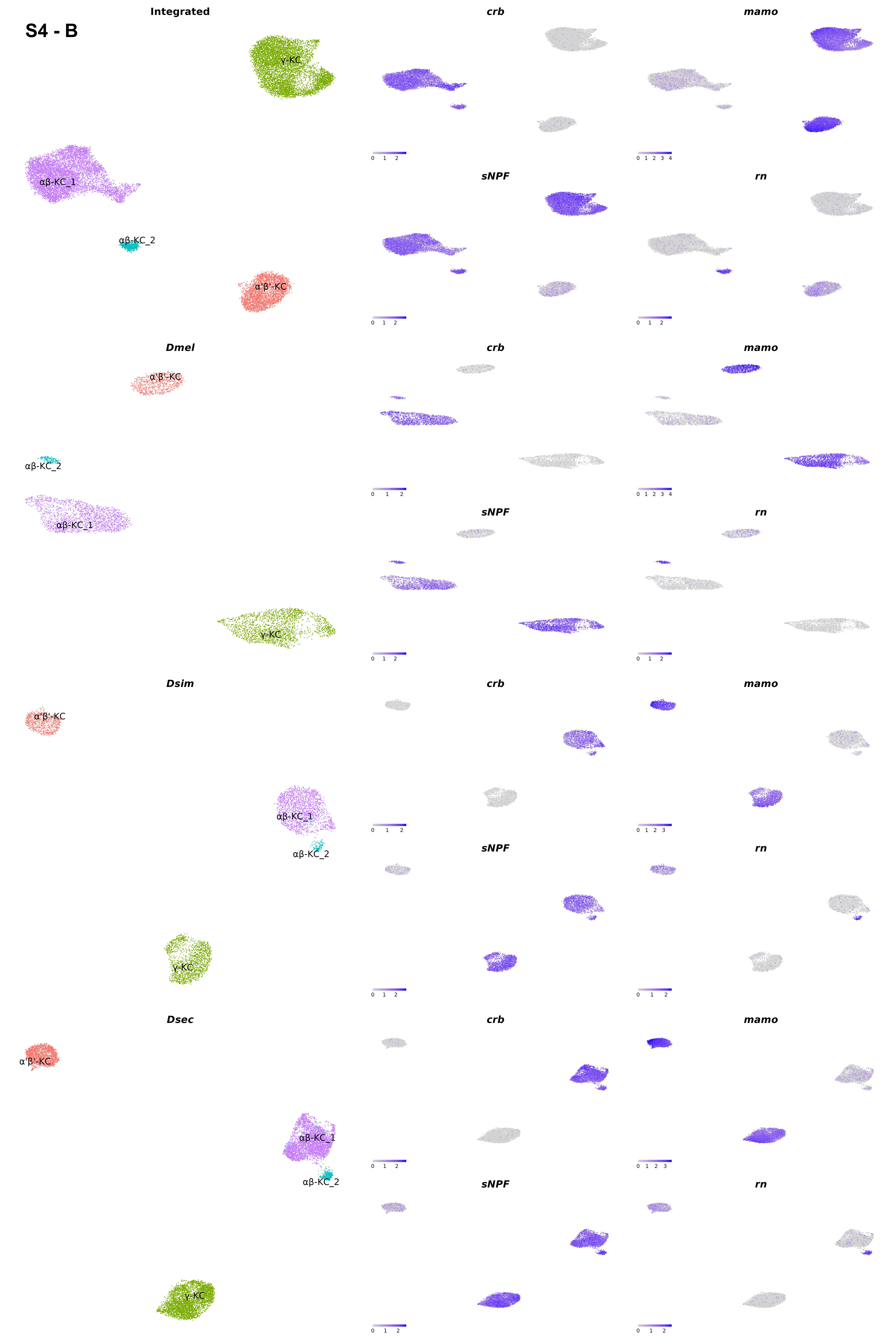
S4 - B
Integrated
γ-KC
αβ-KC_1
αβ-KC_2
α'β'-KC
crb
mamo
sNPF
rn
Dmel
α'β'-KC
αβ-KC_2
αβ-KC_1
γ-KC
crb
mamo
sNPF
rn
Dsim
α'β'-KC
αβ-KC_1
αβ-KC_2
γ-KC
crb
mamo
sNPF
rn
Dsec
α'β'-KC
αβ-KC_1
αβ-KC_2
γ-KC
crb
mamo
sNPF
rn

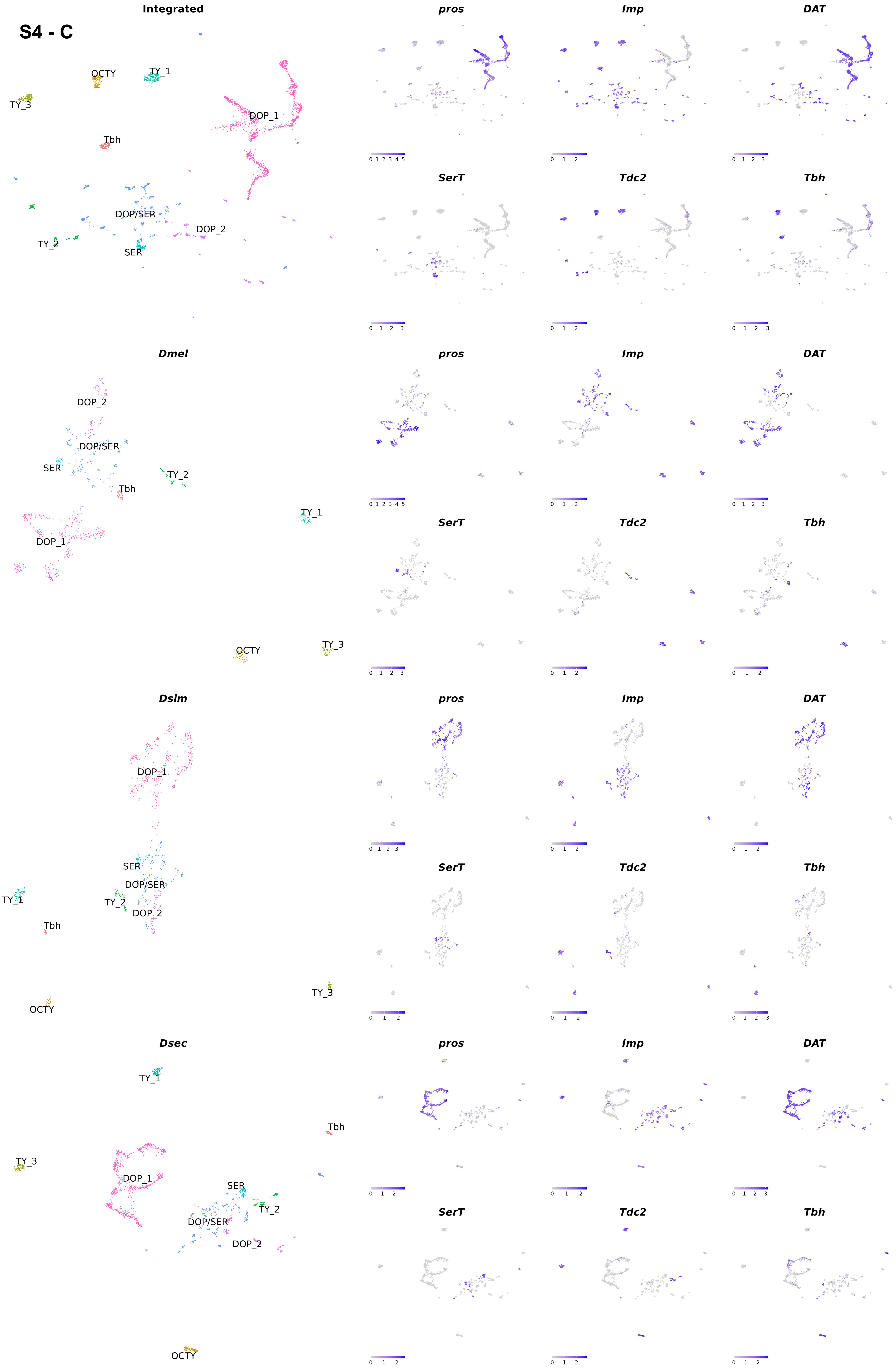
S4 - C
Integrated
OCTY
TY_1
TY_3
DOP_1
Tbh
DOP/SER
DOP_2
TY_2
SER
pros
Imp
DAT
SerT
Tdc2
Tbh
Dmel
Dsim
Dsec

S4 - D

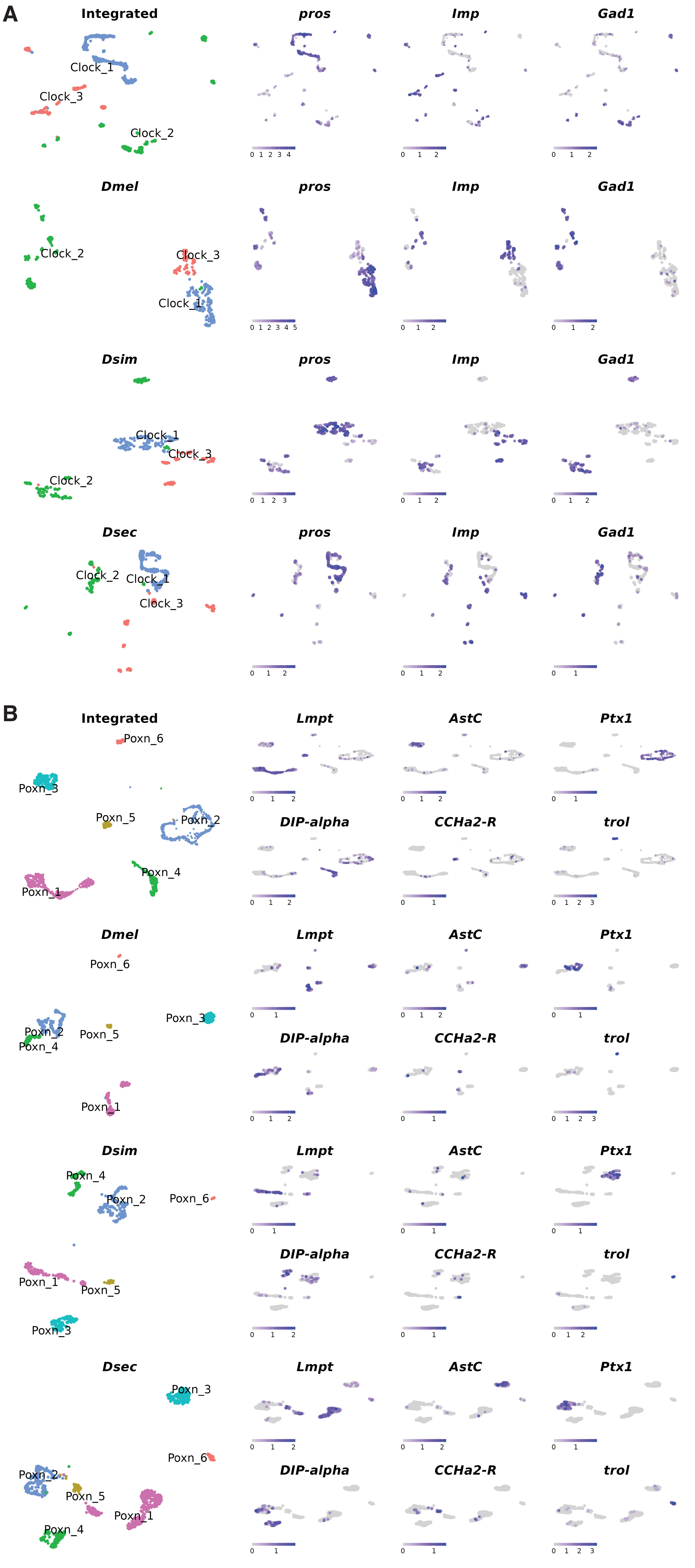
A
Integrated
pros
Imp
Gad1
Clock_1
Clock_3
Clock_2
Dmel
Dsim
Dsec
B
Lmpt
AstC
Ptx1
DIP-alpha
CCHa2-R
trol
Poxn_1
Poxn_2
Poxn_3
Poxn_4
Poxn_5
Poxn_6

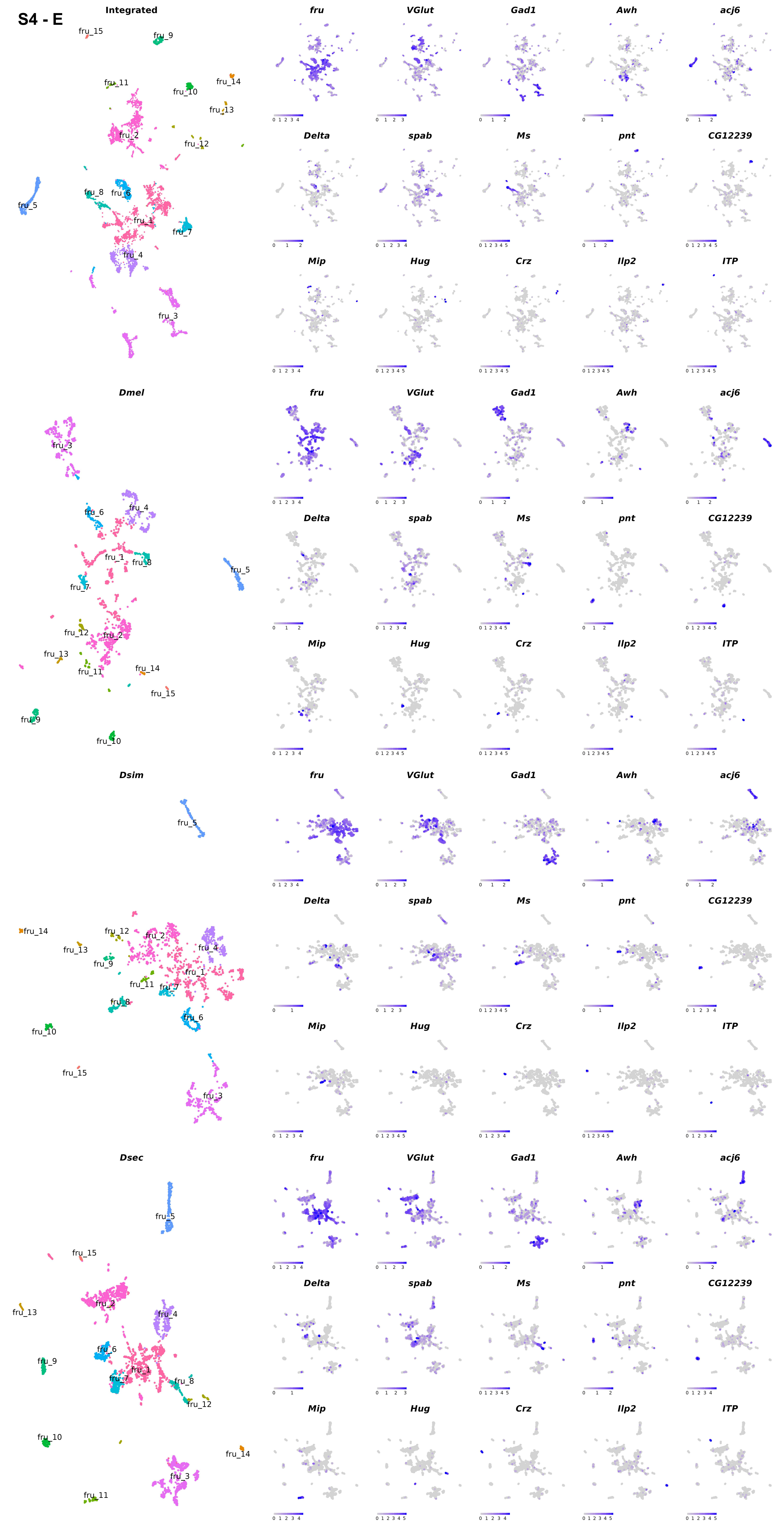

S4 - E
Integrated
fru_15
fru_9
fru_11
fru_14
fru_10
fru_13
fru_2
fru_12
fru_5
fru_8
fru_6
fru_1
fru_7
fru_4
fru_3
fru
VGlut
Gad1
Awh
acj6
Delta
spab
Ms
pnt
CG12239
Mip
Hug
Crz
Ilp2
ITP
Dmel
fru_3
fru_6
fru_4
fru_1
fru_8
fru_5
fru_7
fru_12
fru_2
fru_13
fru_11
fru_14
fru_15
fru_9
fru_10
fru
VGlut
Gad1
Awh
acj6
Delta
spab
Ms
pnt
CG12239
Mip
Hug
Crz
Ilp2
ITP
Dsim
fru_5
fru_14
fru_12
fru_2
fru_13
fru_4
fru_9
fru_1
fru_11
fru_7
fru_8
fru_6
fru_10
fru_15
fru_3
fru
VGlut
Gad1
Awh
acj6
Delta
spab
Ms
pnt
CG12239
Mip
Hug
Crz
Ilp2
ITP
Dsec
fru_5
fru_15
fru_2
fru_13
fru_4
fru_6
fru_9
fru_1
fru_7
fru_8
fru_12
fru_10
fru_14
fru_3
fru_11
fru
VGlut
Gad1
Awh
acj6
Delta
spab
Ms
pnt
CG12239
Mip
Hug
Crz
Ilp2
ITP

S4 - F

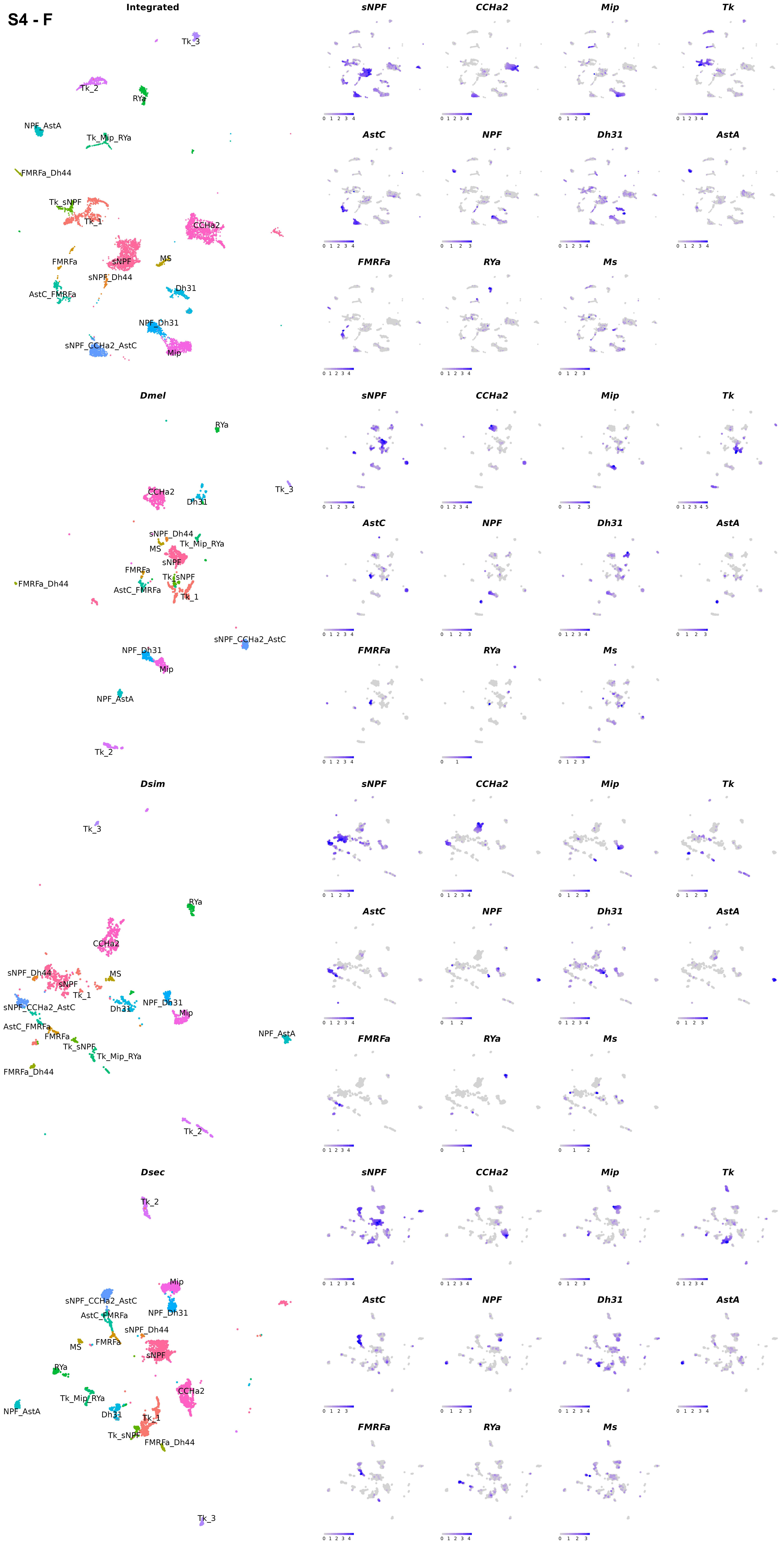
Integrated
Tk_3
Tk_2
RYa
NPF_AstA
Tk_Mip_RYa
FMRFa_Dh44
Tk_sNPF
Tk_1
CCHa2
FMRFa
sNPF
MS
sNPF_Dh44
AstC_FMRFa
Dh31
NPF_Dh31
sNPF_CCHa2_AstC
Mip
sNPF
CCHa2
Mip
Tk
AstC
NPF
Dh31
AstA
FMRFa
RYa
Ms
Dmel
RYa
CCHa2
Dh31
Tk_3
sNPF_Dh44
Tk_Mip_RYa
MS
sNPF
FMRFa
Tk_sNPF
FMRFa_Dh44
AstC_FMRFa
Tk_1
sNPF_CCHa2_AstC
NPF_Dh31
Mip
NPF_AstA
Tk_2
sNPF
CCHa2
Mip
Tk
AstC
NPF
Dh31
AstA
FMRFa
RYa
Ms
Dsim
Tk_3
RYa
CCHa2
sNPF_Dh44
MS
sNPF
Tk_1
sNPF_CCHa2_AstC
Dh31
NPF_Dh31
Mip
AstC_FMRFa
FMRFa
NPF_AstA
Tk_sNPF
Tk_Mip_RYa
FMRFa_Dh44
Tk_2
sNPF
CCHa2
Mip
Tk
AstC
NPF
Dh31
AstA
FMRFa
RYa
Ms
Dsec
Tk_2
Mip
sNPF_CCHa2_AstC
NPF_Dh31
AstC_FMRFa
sNPF_Dh44
MS
FMRFa
sNPF
RYa
CCHa2
Tk_Mip_RYa
NPF_AstA
Dh31
Tk_1
Tk_sNPF
FMRFa_Dh44
Tk_3
sNPF
CCHa2
Mip
Tk
AstC
NPF
Dh31
AstA
FMRFa
RYa
Ms

S4 - G

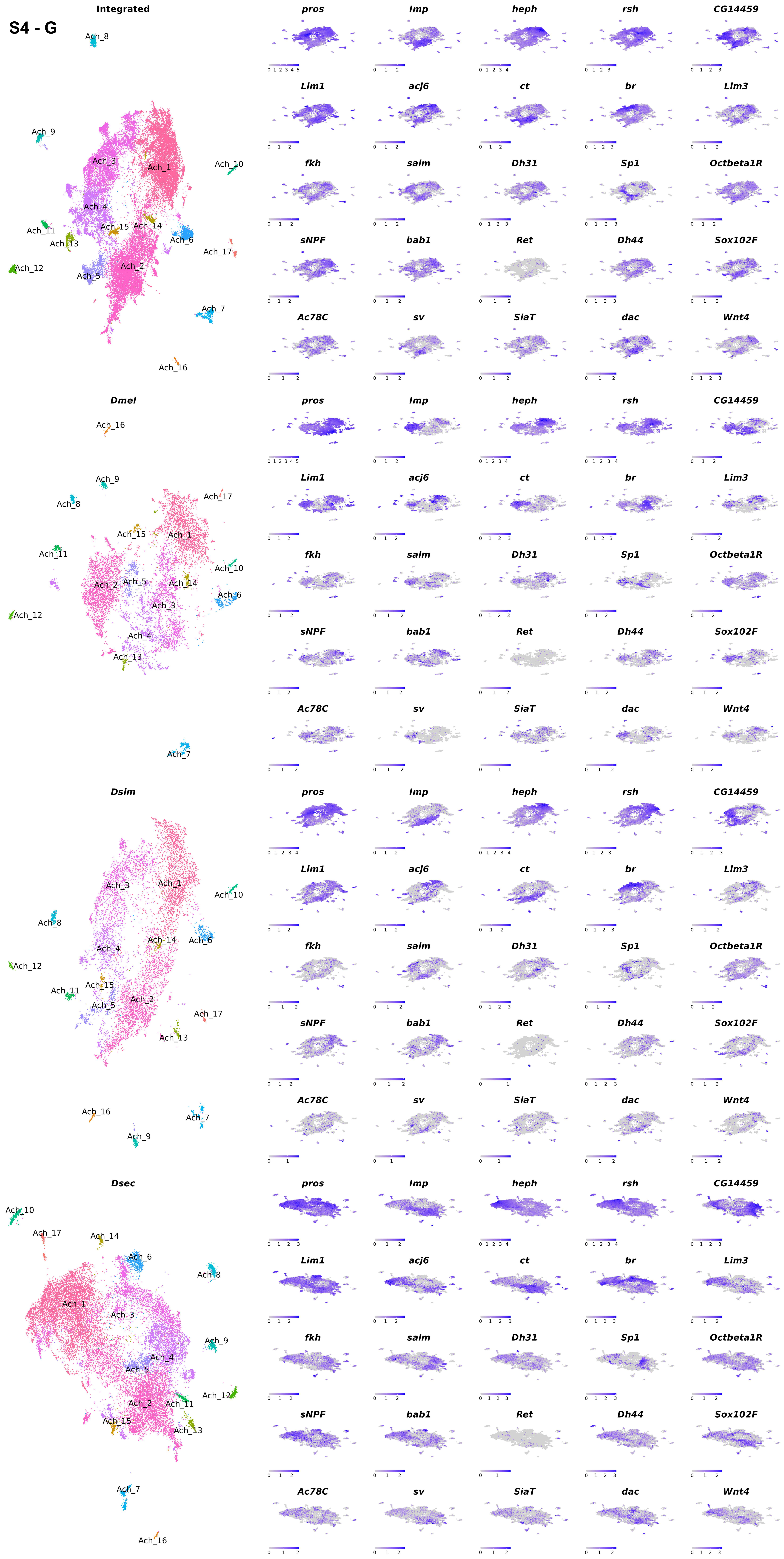

Integrated
Ach_1
Ach_2
Ach_3
Ach_4
Ach_5
Ach_6
Ach_7
Ach_8
Ach_9
Ach_10
Ach_11
Ach_12
Ach_13
Ach_14
Ach_15
Ach_16
Ach_17
Dmel
Dsim
Dsec
pros
Imp
heph
rsh
CG14459
Lim1
acj6
ct
br
Lim3
fkh
salm
Dh31
Sp1
Octbeta1R
sNPF
bab1
Ret
Dh44
Sox102F
Ac78C
sv
SiaT
dac
Wnt4

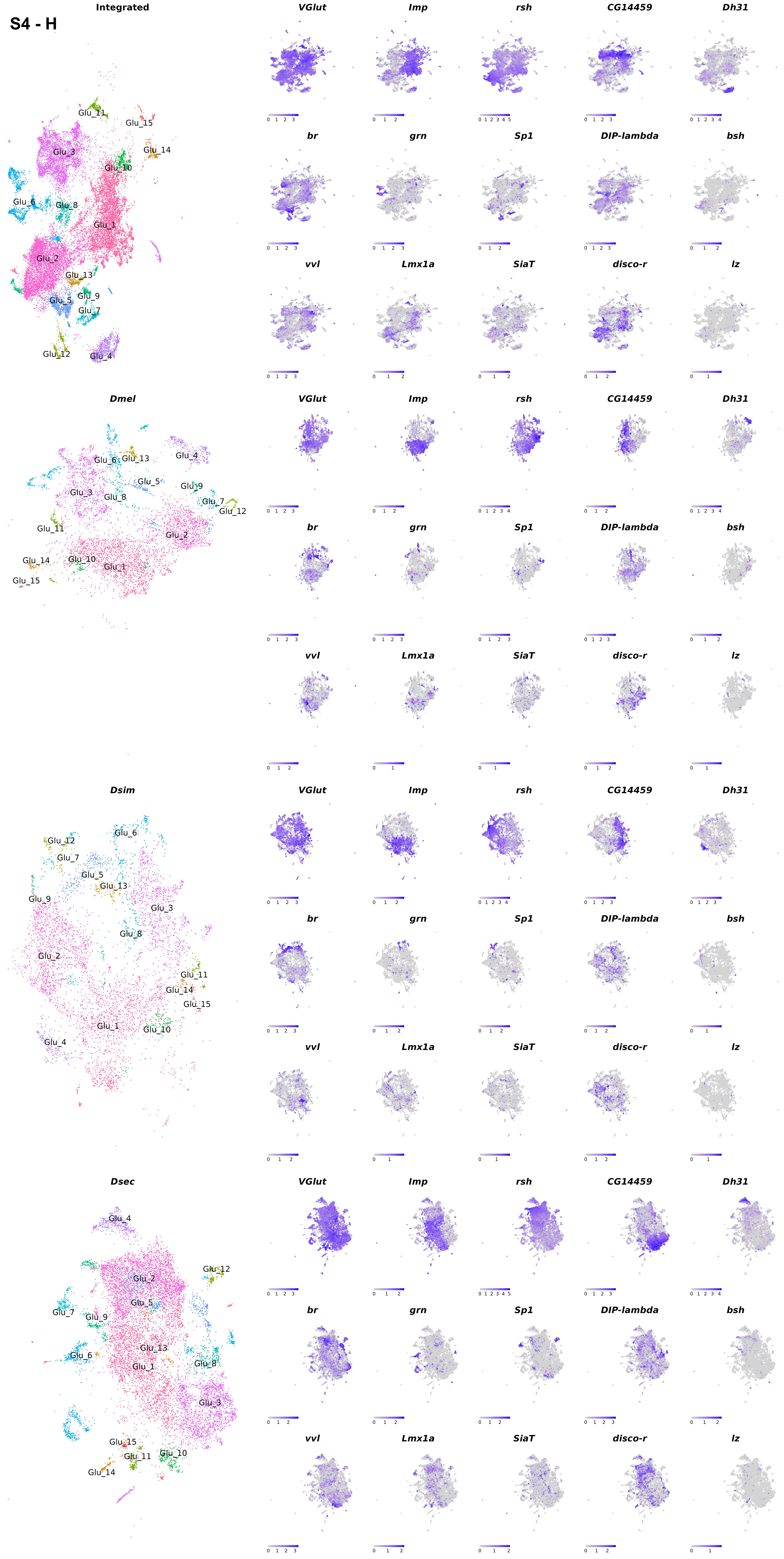
S4 - H
Integrated
Dmel
Dsim
Dsec
VGlut
Imp
rsh
CG14459
Dh31
br
grn
Sp1
DIP-lambda
bsh
vvl
Lmx1a
SiaT
disco-r
lz
Glu_1
Glu_2
Glu_3
Glu_4
Glu_5
Glu_6
Glu_7
Glu_8
Glu_9
Glu_10
Glu_11
Glu_12
Glu_13
Glu_14
Glu_15

S4 - I

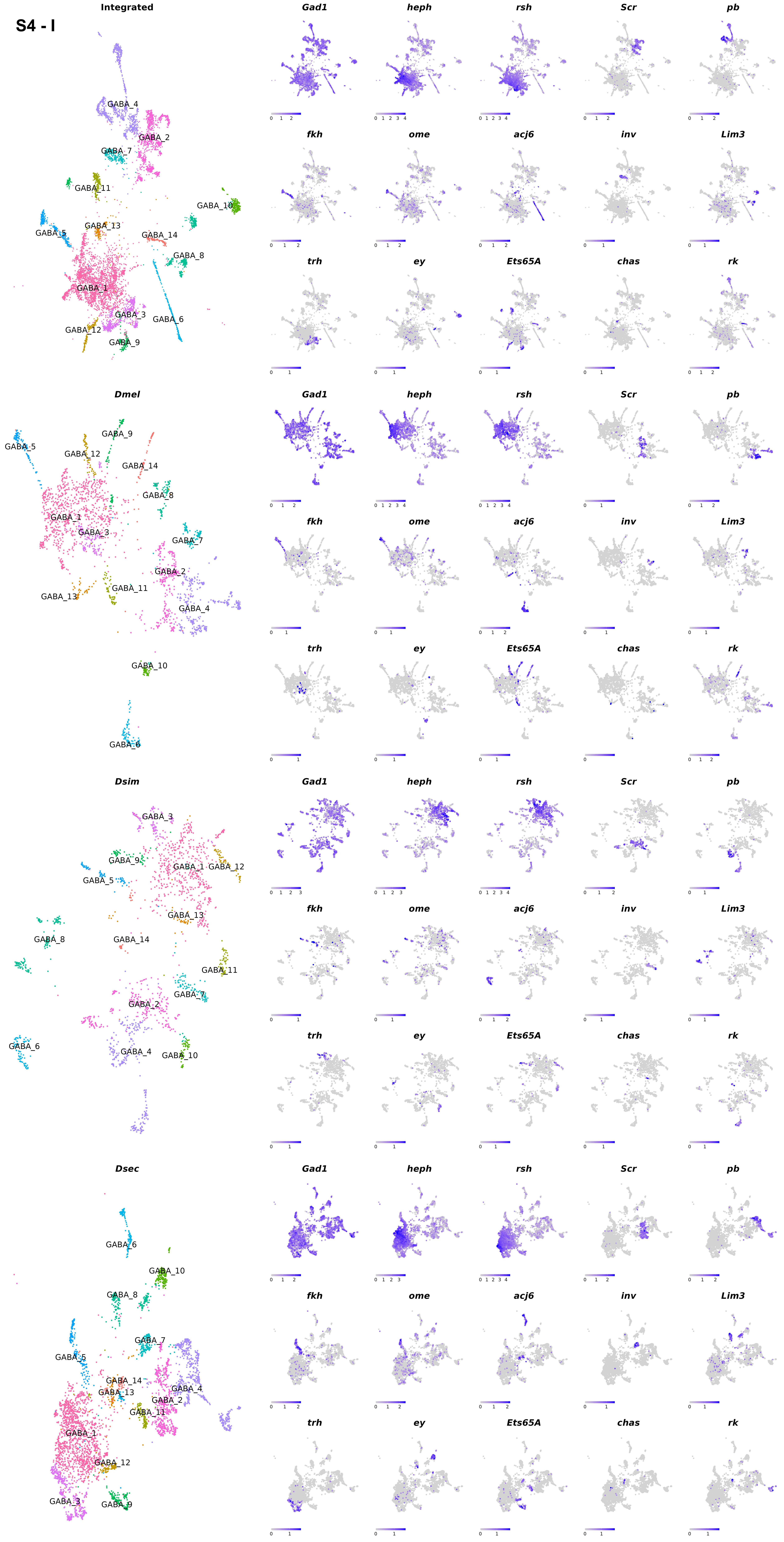
Integrated
Dmel
Dsim
Dsec
Gad1
heph
rsh
Scr
pb
fkh
ome
acj6
inv
Lim3
trh
ey
Ets65A
chas
rk
GABA_1
GABA_2
GABA_3
GABA_4
GABA_5
GABA_6
GABA_7
GABA_8
GABA_9
GABA_10
GABA_11
GABA_12
GABA_13
GABA_14
