# Supplementary material for: Comparative single-cell transcriptomic atlases of drosophilid brains suggest glial evolution during ecological adaptation
Source: PLoS Biol. 2025 Apr 29;23(4):e3003120. doi: 10.1371/journal.pbio.3003120 (PMC12040179; doi:10.1371/journal.pbio.3003120)
Supplement: Attachment — Submitted filename: 250125_Lee_RespRev.pdf [file pbio.3003120.s025.pdf]

PBIOLOGY-D-23-03338R1: RESPONSE TO REVIEWERS

We thank the editors and reviewers for their careful reading and constructive criticisms of our manuscript. Below, we provide responses to each of the raised issues. We first note that, following important technical concerns expressed by several reviewers (e.g., mapping of *D. sechellia* reads to the *D. simulans* genome, and integration of single-cell datasets of different species) we have completely re-worked our analysis pipeline, including mapping of each species' reads to their respective genomes. This effort necessarily has meant a revision of the plots in all of the figures; while some details have (unsurprisingly) changed, as described below, the main conclusions of the study remain supported by our analyses.

<u>Reviewer #1:</u>

This ms addresses the question of brain evolution with three species of fruit flies, the workhorse Dmel, Dsim and Dsech. These were carefully chosen to provide a good phylogenetic framework for interpretation, with the highlight on Dsech, a specialist to contrast with the two generalists. Dsim and Dsech are comparably evolutionarily diversed from Dmel in terms of time. Of course the work benefits immensely from the extremely well annotated Dmel genome.

The ms uses comparative snRNAseq as the one and only research tool, as a modern alternative to comparative neuroanatomy.

Results are as predicted: more differences in Dsech relative to the other species. More surprising, perhaps is the finding of overall very high levels of similarity in sn gene expression across the species. Similarity is used to infer evolutionary conservation—see comment on this below.

A nice addition was to rear Dsech on standard medium and then on medium supplemented by its sole natural host; a few changes in gene expression were observed but mostly stasis. As this was the only manipulation performed in a study with a species (Dmel) that is manipulated more than it is described, this was noteworthy.

The methods are straightforward, using some of the best single cell RNAseq analytics approaches.

The paper is an information-dense descriptive analysis that should provide a foundation for follow up work on brain evolution. The authors are appropriately circumspect about causality vs. correlation. It would be great to see creative manipulations of gene expression in Dsim to see if a preference for Dsech could be induced.

Very scholarly treatment, engaging in some of the big discussion points in evolutionary neuroscience.

I have the following concerns—all relatively minor.

As with all studies of this young genre, there is an issue of how well can one classify

cells based solely on expression patterns. As noted in an influential paper by David Schulz and colleagues, reliable classification occurs only with a combination of molecular and anatomical information, i.e., combining molecular cell type and anatomical cell type. This reviewer is not asking for a re-analysis, but it would be good to mention this concern as a caveat.

RESPONSE: We thank the reviewer for this insightful comment. We acknowledge the inherent limitations of relying solely on gene expression data from single-nucleus RNA-sequencing for cell-type classification. As they noted, additional anatomical information is crucial for fully validating cell identities. In our manuscript, we now explicitly address this caveat in the Discussion section as follows (including citing of the paper mentioned by the reviewer):

"*We could classify 107 cell types in our atlases, though note that gene expression patterns alone are not necessarily sufficient to distinguish cell types within or between species [54], which will require future developmental and anatomical analyses*."

Re "similarity" = conservation. Perhaps the standard lab environment is so limiting that species-specific differences are not induced. This certainly has been seen in considering the effects of genetic mutations on phenotype in mice, comparing standard impoverished lab environment vs an enriched environment. That is to say, the similarity = conservation conclusion is reasonable, but an alternative also exists that should be addressed.

RESPONSE: We thank the reviewer for raising this important consideration. We acknowledge that the standardized laboratory environment might mask interspecific variation that would be observed under more diverse or enriched conditions. However, our and others' previous studies (*Shahandeh et al. Nature 2024; Dürr et al. bioRxiv 2024*) have shown that these species display numerous phenotypic differences in behavior, neuroanatomy and physiology despite their growth under the same culture conditions. Thus the differences we report here on the cellular composition and gene expression patterns must reflect brains capable of producing species-specific phenotypes at the whole animal level. Our observation that some aspects, such as neuronal composition, are so similar is perhaps unexpected but nevertheless informative for thinking about how species-specific behaviors arise. Future studies of these drosophilids' brains under other conditions is likely to reveal additional differences between species. We refer to these issues in the Discussion:

"Finally, we acknowledge that our atlases almost certainly do not capture the full extent of interspecific differences in gene expression. Although several lab-based studies of the peripheral olfactory system identified several olfactory sensory neuron populations that are increased or decreased in D. sechellia compared to the other species [32,33,40,53], it is possible that some interspecific variation in the brain remains cryptic under these controlled conditions. Additional experiments under more natural ecological contexts could reveal other brain-related adaptations."

The proportion of genes captured with this sn protocol appears lower than what one sees in other studies. If these are the most highly expressed or most stably expressed genes, they might be giving an overly conserved perspective.

RESPONSE: We appreciate the reviewer's concern regarding the potential bias introduced by our snRNA-seq protocol capturing fewer genes overall, potentially skewing our dataset toward more highly expressed or more conserved genes. We acknowledge this technical limitation (which applies to the vast majority of snRNA-seq studies in the literature; our protocol is essentially identical to that used for the Fly Cell Atlas (*Li et al. Science 2022*)), but our analyses suggest that this bias is not overly restrictive. First, if our approach was capturing only highly-expressed, conserved genes, we would expect the most highly-expressed genes to show diminished interspecific differences. However, as shown in Figure 3, when we focus on the 50 most highly-expressed genes, we do not observe a consistent pattern of reduced divergence among these genes. Specifically, in Figure 3A, the data points towards the right of each plot (i.e., the more highly-expressed genes) are not closer to the smoothed line. Second, our differential expression (DEG) analyses show that broadly expressed genes can also exhibit cell-type-specific differences across species. For example, as described in the Results section (lines 236-244), among 593 DEGs from the *Dmel-Dsec* pair, 290 genes (48.9%) were identified from a single cell type, and 459 genes (77.4%) were identified from no more than 3 cell types, even though the majority of these genes are broadly expressed (on average, expressed in 79/107 cell types in *D. melanogaster*, minimum percent expression threshold: 5%). In other words, even though our method may preferentially retain highly expressed transcripts, we still detect substantial interspecific variation among these genes.

Together these findings reinforce our conclusion that the conserved and divergent features we report are not merely artifacts of focusing on highly expressed genes. We also refer to these issues in the Discussion:

"In addition, as technical limitations of scRNA-seq currently constrain our ability to detect changes in rare cell types or genes with low expression, future deeper sequencing efforts and larger-scale single-cell atlases might reveal further differences."

In the Discussion, the opening implies that this study examined how brains evolve. I would argue that there is no "how" here, just snapshots of expression showing differences/similarities. That's different in how those came to be. That would require experimental evolution. That said, the present study certainly addresses this big and important question, providing key observations that could be the basis of experimental evolution studies.

RESPONSE: We thank the reviewer for this comment and rephrased the opening sentence as follows:

From: "*Our work provides an unprecedented comprehensive view of how animal brains evolve in a well-defined phylogenetic and ecological framework, by taking advantage of the relatively small brains of closely-related drosophilid species to generate and compare whole central brain cellular atlases*."

To: "*Taking advantage of the small brains of closely-related drosophilid species, our work provides an unprecedented, comprehensive view of the molecular and cellular*

conservation and divergence of animal brains in a well-defined phylogenetic and ecological framework."

Reviewer #2:

In this manuscript, Lee and Benton perform a comparative transcriptomic analysis of a trio of closely related drosophilids that have a very distinct phylogenetic and ecologic relationship: D. simulans (Dsim) and D. sechelia (Dsec), which are sister species (diverged only ~0.2 million years ago) and D. melanogaster (Dmel), which diverged from the Dsim/Dsec clade ~4 million years ago. In terms of ecological niches, Dmel and Dsim are generalists, while Dsec is specialized on feeding on noni fruit. While the Benton lab has studied in the past the role of sensory neurons in this specialization, the authors here focus on the central nervous system and compare the neuronal composition of the central brain of these species.

To do this, they use single-nucleus mRNA sequencing to read the transcriptomes of ~45,000-50,000 cells from each drosophilid central brain coming from six independent biological replicates. After integrating these datasets, they found the overall composition of the central brains to be fairly conserved. They also identify a number of conserved gene expression profiles between cell types/clusters of the different drosophilids, which provide as a resource of cell type molecular fingerprints. They then address the more pertinent question, i.e. what are the differences in cell type composition in the drosophilid trio with a particular interest in how the noni specialist Dsec is different from the two generalists. In this, they are aided by the strategic selection of the species, which allows them to compare a duo of generalists (Dmel and Dsim) and a duo of a generalist and a specialist (Dmel and Dsec) that diverged at the same time (3-4 million years ago). Interestingly, most of the differences they find are in glial cells:

A) Dsec has proportionally more perineurial glial cells than Dmel and Dsim.

B) Four out of the five glial cell types show an accelerated rate of gene expression change in the noni specialist drosophilid (Dsec) than in the generalist (Dsim)

C) They find forty differentially expressed genes in the Dsec glial cell types, which is consistent with this increased divergence in gene expression

D) Finally, when they compare the transcriptome of Dsec central brain neurons in the presence or absence of noni juice paste in the food, they find four out of the six differentially expressed genes to be differentially expressed in glial cells.

The above observations allow them to propose a model where the glial cells were the first to respond to the presence of noni fruit in their diet and they were subsequently evolutionarily reshaped to allow Dsec to adapt to this very specific niche.

Overall, it is a very interesting paper bringing forward a number of ideas that would not be able to be drawn from other experimental systems. It is of broad interest and it would be a very useful part of the literature. However, I have a number of comments that the authors could consider to make the conclusions of the paper more robust.

- I am still not very convinced by the use of single-cell sequencing data integration to identify differences in cell type composition. These algorithms are meant to integrate samples that are similar and may "force" different cell types to

integrate. Did the authors try different algorithms or different parameters to integrate? How did they evaluate the quality of the integration? This information would be useful to be in the Methods. Moreover, I think that it would be useful for the authors to compare the three atlases without integrating. Do they find one-to-one correspondences between different dataset clusters based on marker gene expression?

RESPONSE: The reviewer raises an important point about the challenges of integrating single-cell datasets of distinct species to identify differences in cell-type composition; there are still only a few studies in the literature that compare species in this way so there is no unified method for performing or assessing such analyses. To address this issue in our work, we re-established our analysis pipeline and, most importantly, compared the one-to-one correspondence of cell clusters between the integrated atlas and each of the three non-integrated atlases (Supplementary Figure 4). In all of the 107 classified cell types we validated that the co-clustering of cells from different species reflects their distinct identities in the individual species' atlases i.e., there was no "forcing" of cell-type integration in the integrated atlas. We have expanded the Methods section to detail these procedures (and also included a summary flowchart in Supplementary Figures 1 and 2). All marker-gene based comparisons among integrated and single-species atlases are shown in Supplementary Figure 4.

- On a similar note, the difference in frequencies of homologous cell types should be better supported. First, there are different algorithms that try to evaluate such differences in a way that is tailored to single-cell sequencing data, such as miloR from the Marioni lab. Second, some of the differences the authors find could be a result of technical differences, e.g. in nuclear sorting. It would be worth if the authors could verify some of these differences in situ. For example, the difference in the number of perineurial glial cells should be fairly easy to capture by immunohistochemistry or FISH.

RESPONSE: We appreciate the reviewer's concern regarding our cell-type frequency analysis. We attempted to implement MiloR as an alternative method; however, due to the high complexity of the central brain dataset - where we have classified 107 cell types - we found MiloR to be less powerful in resolving cell types than our current approach. In line with the reviewer's suggestion, we performed *in vivo* validation experiments for perineurial glial cells using both FISH and immunohistochemistry. These experiments confirmed that the perineurial glial cell signal (and overall glial counts) is indeed reduced in *D. sechellia*. We have included these new results in the manuscript (Figure 2D-E and Supplementary Figure 9).

We further note that in an independent snRNA-seq project in our lab (Mermet *et al.*, *2025*) of antennal neurons in *D. melanogaster*, we have found a very strong correlation between cell representation in the snRNA-seq cell atlas and numbers of neurons quantified *in situ* (Reviewer Figure 1). This observation gives us confidence that cell representation in snRNA-based atlases are highly quantitative. Brains and antennae are of course different tissues, but extraction of nuclei is, if anything, likely to be more technically challenging in the latter given antennal neurons are encased in cuticle.

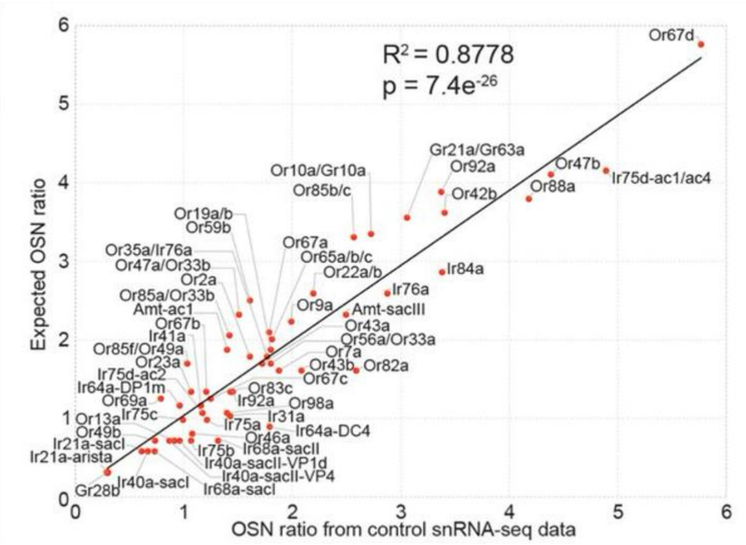

Reviewer Figure 1 (from Mermet et al. 2025 doi:10.1101/2025.01.16.632932, Figure 1H) | Scatter plot of the relative abundance of each neuronal population in an antennal developmental atlas with their relative abundance as quantified *in situ*.

Also, regarding the difference in frequencies, do the authors really believe that the number of clock neurons is affected when Dsec is grown with or without noni fruit?

RESPONSE: With the revised single-cell atlases (taking into account this reviewer's valuable technical concerns), we noted that the small Clock cell type frequency difference originally reported is no longer observed (Supplementary Fig. 12). We have revised the Results section accordingly.

- The authors use different cutoffs at different parts of the paper that are not justified. For example, in the section "Conserved gene expression patterns in drosophilid central brains" they select genes that are expressed in "at least 5% of Dmel cells". Then, they select genes that are expressed in over 30% of cells across species and focus on genes that are restricted to 1-9 clusters, etc. This is also observed in other parts of the paper. I am wondering how the authors came up with these cutoffs and whether the use of different cutoffs could have affected some of their conclusions.

RESPONSE: We appreciate the reviewer's concern, and we acknowledge that the thresholds used for our analyses are arbitrary. Each threshold that we chose was to address a specific question, and served as practical cutoffs to highlight a subset of genes with robust or well-conserved expression patterns, thereby providing an informative resource for future studies. To offer readers the flexibility to adjust these thresholds, for example with stricter cut-offs, we have provided datasets in Supplementary Tables 3 and 4, which detail the percent of cells expressing each gene and correlation coefficients. Of the analyses presented, the Gene Ontology (GO) term analysis (Supplementary Figure 5C) is potentially the most sensitive to variations in these thresholds. However, we tested an alternative Spearman's ρ cutoff (adjusting from 0.7 to 0.6 or 0.8) and observed that our conclusions remained consistent (see Reviewer Figure 2 on the next page).

$\rho > 0.6$

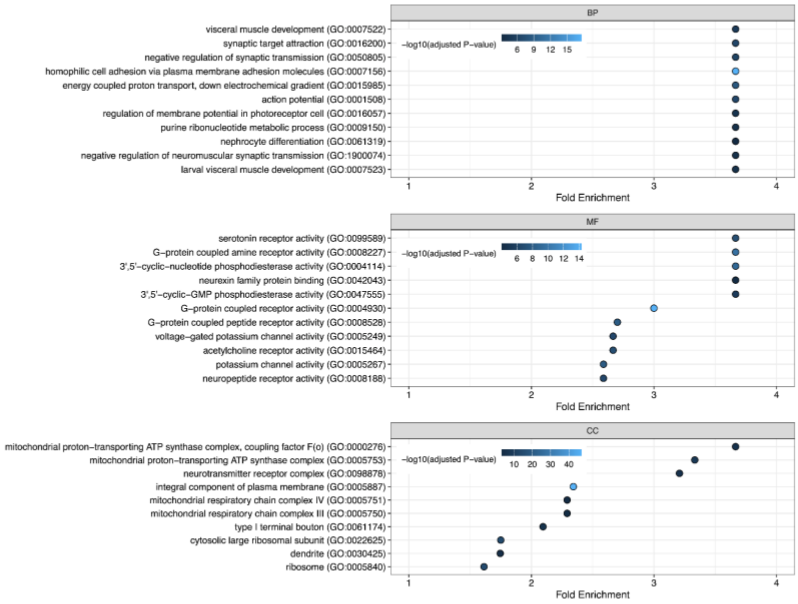

$\rho > 0.7$

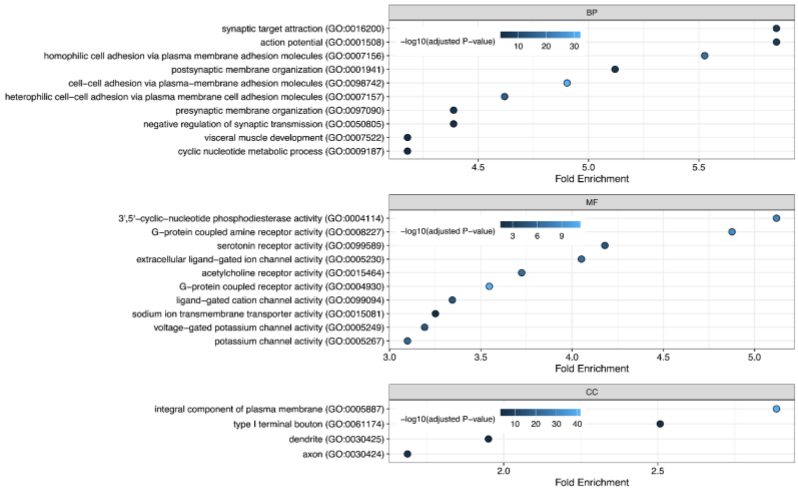

$\rho > 0.8$

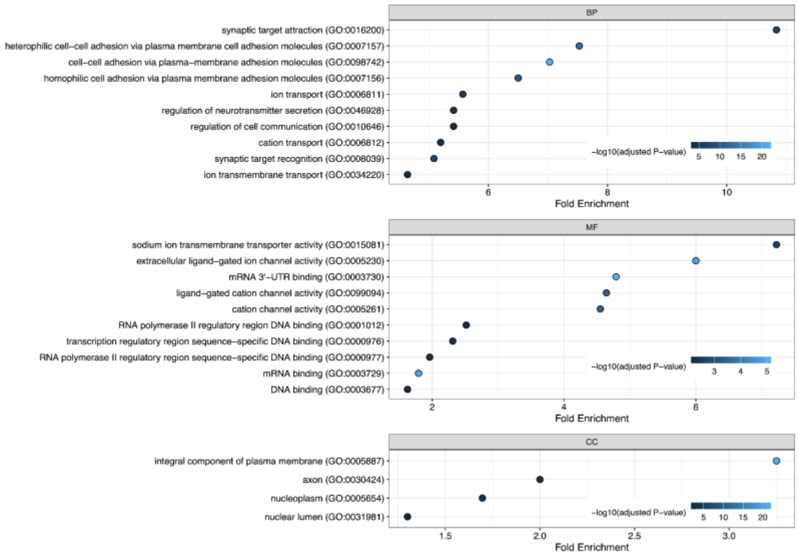

Reviewer Figure 2 | GO enrichment analyses for 656 (top, $\rho > 0.6$), 413 (middle, $\rho > 0.7$) and 185 (bottom, $\rho > 0.8$) genes with highly conserved expression patterns across all three pairwise comparisons.

- Finally, I am also concerned by the mapping of Dsec reads on the Dsim genome. This creates an imbalance between the two species (as Dsim is mapped in each own genome, while Dsec is mapped on a sister species genome). Since the entire study is based on the strategic selection of the drosophilid trio to dissect the differences in gene expression that are caused by adaptive versus neutral evolution, this imbalance may skew the results and the interpretation. Assuming that generating and annotating a reliable Dsec genome is challenging, wouldn't it be better that the authors do all their analyses with all the datasets mapped to the Dmel genome (i.e. Dmel-to-Dmel vs Dsim-to-Dmel vs Dsec-to-Dmel)?

RESPONSE: This is an excellent point. Our original mapping efforts were performed at a time when a high-quality annotated *D. sechellia* genome was not available. For the revised manuscript, we have re-performed our entire analysis using the updated *D. simulans* and *D. sechellia* reference genomes and annotations, thereby avoiding any potential skew from mapping *D. sechellia* reads to the *D. simulans* genome. Additionally, as in our original manuscript, we repeated the gene expression analysis using a single *D. melanogaster* reference genome. To be as conservative as possible, we only considered genes to be differentially expressed if they were confidently identified by both alignment strategies i.e., species-to-own genome mapping and species-to-*D. melanogaster* genome mapping. The overall conclusions are the same as in the previous analyses with *Dsec*-to-*Dsim* mapping.

<u>Reviewer #3:</u>

Lee & Benton use single-nuclei sequencing of the central brain of three drosophilids to present the first overview of brain evolution at the single cell level. The authors examine changes in cell type number and gene expression divergence between two generalist species and the feeding specialist D. sechellia. The authors find that cell types overall are highly conserved between these species, but that glial cell types on average exhibit greater between-species divergence than neuronal cell types. Divergence at the neuronal level is concentrated in two peptidergic cell types. Gene expression and cell type divergence occur faster on the branch leading to D. sechellia than to the generalist species D. simulans. This paper is a landmark in the field and is of broad interest. I have several suggestions to improve or strengthen the interpretations and analyses.

Major suggestions:

A significant concern is the interpretation of the increased divergence in D. sechellia as adaptation to its niche. D. sechellia has a much smaller population size than D. simulans which is sufficient to explain the increased divergence without adaptation. The authors could specifically test for signatures of gene expression adaptation to support their adaptation conclusion. The authors could also note the alternative possibility of reduced purifying selection relative to mutation and drift which would increase divergence in small population sizes such as D. sechellia. Indeed, this could result in increased compensatory changes in gene expression, of which several were observed in D. sechellia in this study.

RESPONSE: We acknowledge that the smaller population size of *D. sechellia* might reduce purifying selection, potentially leading to more frequent gene expression

changes driven by drift (not adaptation); moreover, some of these changes might be compensated by additional expression shifts that further exacerbate divergence in gene expression profiles between species. We have now mentioned these possibilities in the Discussion. While we agree that testing for signatures of gene expression adaptation would be valuable, a relevant analysis necessitates broader phylogenetic sampling across multiple *Drosophila* species, which lies beyond the scope of our current study. We anticipate this will be an important avenue for future research. We edited the Discussion section as follows:

From: *"The expectation, under neutral conditions, is for D. simulans and D. sechellia to exhibit a similar degree of gene expression divergence when compared to D. melanogaster. However, our data revealed more pronounced shifts in brain gene expression in D. sechellia, suggesting this lineage underwent non-neutral gene expression changes, possibly due to its adaptation to its unique ecological niche. Glia stand out as the cell types displaying the most potential adaptive expression alterations, reinforcing the notion that they have an important contribution to brain evolution. Among the genes displaying altered expression patterns, those associated with metabolism are particularly enriched. The metabolic gene regulatory network likely underwent remodeling to adapt to the new nutritional conditions of the species' unique niche."*

To: *"Under neutral conditions, the expectation is for D. simulans and D. sechellia to exhibit a similar degree of gene expression divergence when compared to D. melanogaster. However, our data revealed more pronounced shifts in brain gene expression in D. sechellia, suggesting that this lineage underwent non-neutral gene expression changes, possibly due to its adaptation to its unique ecological niche. Glia stand out as the cell types displaying the most potential adaptive expression alterations, reinforcing the notion that they have an important contribution to brain evolution. Among the genes displaying altered expression patterns, those associated with metabolism are particularly enriched. The metabolic gene regulatory network likely underwent remodeling to adapt to the new nutritional conditions of the species' unique niche. An alternative hypothesis, however, is that D. sechellia's smaller effective population size [60] reduces the efficacy of purifying selection, allowing neutral or slightly deleterious mutations to accumulate and greater expression divergence. Furthermore, the observed inverse expression changes in certain paralog pairs in D. sechellia (e.g., CG9394/CG18135 and CAH2/CAH3) suggest potential compensatory mechanisms that might further exacerbate divergence in gene expression profiles. Future analyses of signatures of selection on gene expression with broader phylogenetic sampling will be key to distinguishing adaptive and non-adaptive scenarios."*

One of the major conclusions of the paper is that glial gene expression diverges faster than that of neurons. This is very interesting and additional analyses would strengthen this conclusion. For example, is this analysis sensitive to the number of genes used (I.e. different than 50)? Furthermore, glial cells show larger cell type abundance than most neuronal clusters likely resulting in increased statistical power. It would be important to see whether the gene expression correlations are robust when cell types are subsampled to the same cell numbers. This finding would also be strengthened if the results extend to divergence in principal components and not

just at the gene level.

RESPONSE: We appreciate the reviewer's suggestion to employ principal component analysis (PCA) to further test whether glial cells exhibit faster gene expression divergence than neurons. However, in our cross-species, single-cell dataset PCA is substantially influenced by both interspecies variation and differences among diverse cell types. As a result, the largest principal components tend to separate cell types (e.g., glia vs. neurons) and species (e.g., *D. melanogaster* vs. *D. sechellia*), rather than providing a direct measure of how quickly glial versus neuronal gene expression diverges.

Regarding the number of genes considered in Figure 3B, our re-analysis with the updated pipeline shows that the divergence among glial cell types is not as pronounced as in our initial findings. Nevertheless we confirm that glial cell types display the highest number of differentially expressed genes (DEGs) (Figure 4D); importantly, this is not simply a consequence of their larger cell numbers. Specifically, we observed a correlation between cell population size and DEG counts only when cell numbers were very small (likely due to reduced statistical power), but not for the larger cell populations. As shown in the new Supplementary Figure 10, high DEG counts in glial cell types are not driven solely by their cell-type frequencies. We have updated the Results section accordingly.

There should be some consideration of whether D. sechellia reads can be mapped to the genome of D. simulans without affecting the results. For example, more divergent genes may map less well to D. simulans giving the appearance of lower expression in D. sechellia. This may extend across multiple genes to give the impression of greater overall expression divergence in D. sechellia.

RESPONSE: This is an excellent point. Our original mapping efforts were performed at a time when a high-quality annotated *D. sechellia* genome was not available. For the revised manuscript, we have re-performed our entire analysis using the updated *D. simulans* and *D. sechellia* reference genomes and annotations, thereby avoiding any potential skew from mapping *D. sechellia* reads to the *D. simulans* genome. Additionally, as in our original manuscript, we repeated the gene expression analysis using a single *D. melanogaster* reference genome. To be as conservative as possible, we only considered genes to be differentially expressed if they were confidently identified by both alignment strategies i.e., species-to-own genome mapping and species-to-*D. melanogaster* genome mapping. The overall conclusions are the same as in the previous analyses with *Dsec*-to-*Dsim* mapping.

Minor suggestions:

It seems notable that OPNs show the greatest gene expression divergence between D. melanogaster and the other two species. The authors may want to speculate on the significance of this given the importance of these neurons in the response to noni fruit and other odors.

RESPONSE: The pronounced divergence of OPN gene expression between *D. melanogaster* and the other two species is indeed notable, but as both *D. melanogaster* and *D. simulans* are generalists, this observation suggests that phylogenetic distance rather than ecological differences underlie this pattern. The

underlying reasons for such a strong lineage-specific signal require further investigation and we prefer not to speculate at this stage.

The authors state that 80 genes is too few for GO analysis. While there may be limited power with 80 genes, it does not preclude GO analysis. If no significant GO terms were identified with the 80 genes, this should be stated.

RESPONSE: We thank the reviewer for suggesting a Gene Ontology (GO) analysis of *D. sechellia* DEGs (which is increased to 96 in the revised manuscript). We have conducted this analysis, and the results can be found in the online FlyEnrichr interface (notably under the “Ontologies” tab):

https://maayanlab.cloud/FlyEnrichr/enrich?dataset=0861702930ce911dc279a6e23b3e1efc

However, these results indicated that the most significantly enriched GO terms were frequently supported by only one or two genes. While this does not entirely preclude GO analysis, the small number of genes per term greatly diminishes the power and interpretability of these findings, hence our decision not to emphasize this analysis in our manuscript. We have revised the Results section as follows.

From: *“As the number of DEGs was too limited for traditional GO term enrichment analysis, we manually curated their putative/known functions.”*

To: *“Although we performed GO enrichment analysis on these 96 genes [52], the resulting terms were often supported by only one or two genes (data not shown), limiting interpretability. Therefore, we relied primarily on manual curation to evaluate the putative or known functions of these genes.”*

"We focused on the 1,686 genes that are expressed in at least 5% of D. melanogaster central brain cells.": D. melanogaster Italic font.

RESPONSE: Corrected.

"similar to its known role for enteroendocrine cell specification in the gut [49] Furthermore": add period after [49].

RESPONSE: Corrected.

<u>Reviewer #4:</u>

This study by Lee and Benton generated snRNA-seq atlas data of the central brains from three closely related Drosophilids: Drosophila melanogaster, Drosophila simulans, and Drosophila sechellia. The data quality is good in terms of cell number, expressed genes, and replicates. In the cellular composition level, it was found that all major cell types are well conserved with a few outliers, such as perineurial glia, sNPF and Dh44 neurons. In the gene expression level, it was found that glial cell types showed the greatest divergence between species.

Overall, this study provides a good resource for the fields of species evolution and

comparative neural developmental biology, although there are several studies that have already profiled the central brain, whole brain, and the whole head from Drosophila melanogaster. My major two concerns include the lack of in vivo validation about their key findings on cellular differences and cell-types specific DEGs, as well as the lack of rigor on the clustering analysis.

1. Validation. a) one of the major findings in this study is the identification three cell types in D. sechellia showing different ratios compared with other two species. Perineurial glial cells (PRNs) show a decrease and sNPF and Dh44 neurons show an increase. These ratio differences may come from sampling bias introduced by dissection especially with manual removal of optical lobes, by FACS, or by nucleus filtering steps during data analysis. Considering these factors, it is important to validate these findings with in vivo staining. b). when comparing DEGs among three species, it was found that four types of glial cells exhibited the highest number of DEGs, with unique gene expression changes in D. sechellia. These are interesting findings. Also, when comparing D. sechellia brains between flies raised in normal lab food and in lab food with noni, they found one gene CG5151 is induced by noni food. However, none of these findings are validated with any in vivo assays using RNA in situ, antibody staining, or genetic reporters, which are routine assays for the Benton lab.

RESPONSE: We fully agree with the reviewer about the importance of *in vivo* validation of the predictions from the comparative atlases. We now include such data as summarized below:

1. *Species-specific frequency of Perineurial glial cells (PRNs*): using HCR FISH, we observed a decrease in the expression of the PRN-specific marker, *Tret1-1*, in *D. sechellia*, alongside an overall reduction in the number of glial cells (as assessed by immunofluorescence for the pan-glial marker Repo). These data corroborate our predicted changes in cell-type frequencies (Figure 2D-F). We note that, with our improved analysis pipeline, sNPF(E) population is further divided to two major populations (sNPF and fru_2), and Dh44 population was re-annotated to Ach_10. We found that sNPF and Ach_10 show similar species differences from the previous analysis of sNPF(E) and Dh44; however, both species differences were below the significant threshold from the new results. While we validate only a single population *in situ* in the current manuscript, we note that snRNA-seq atlases are likely to be generally predictive of cell population size *in vivo*, as supported by an independent snRNA-seq project in our lab (Mermet *et al.*, *2025*) of antennal neurons in *D. melanogaster* (see Reviewer Figure 1 above and response to Reviewer 1).

2. *Differentially expressed glial genes***:** through HCR FISH, we confirmed differences in expression of *CAH2* and *ImpE1* in specific glial subtypes, reflecting the cell-type-specific divergence observed in our single-cell analysis (Figure 5C-F). (We attempted analysis of several additional genes, but were unable to detect robust signals by HCR FISH in any species, potentially reflecting that these genes are expressed below the detection threshold of this method and will require future technical developments for *in situ* validation).
3. *Noni-induced gene expression change***:** we used HCR FISH to validate the induced expression of CG5151 when *D. sechellia* was reared on noni-containing food (Figure 6D-E).

Finally, to confirm observed species differences are not from use of specific strains, we performed the *in vivo* validation by HCR FISH in two different strain sets of the drosophilid trio: one that we had sequenced and another one we have used in our prior behavioral studies. Importantly, we obtained the same pattern of results in both strains. These *in vivo* experiments reinforce the robustness of predictions made from our single-cell atlas and indicate that the cell-type frequency and gene expression changes are likely real species differences.

2. Clustering analysis. In Fig 1c, there is a clear D. melanogaster-specific cluster (red) in the center (green and blue tSNE plots showing a hole in the center), and there are also at least two blubs showing either more red cells (Dmel; top part) or more green/blue cells (Dsim/Dse; top and right). However, current analysis pipeline used by the authors could not detect them, which raise concerns for the overall clustering analysis. What are these clusters? What genes and pathways are enriched in these cells? Could these cells be identified as specific clusters with higher clustering resolution? I think these cells are assigned as unannotated (a very big group) so that the sub-type differences will not be revealed. Considering the fact that these unannotated cells take a large fraction (maybe >50% according to fig 1d), I suggest the authors need to pay more attention to them and do further analysis instead of ignoring them by assigning them as unannotated.

RESPONSE: The reviewer raises an excellent point about the nature of the unannotated - and apparently species-specific - cell representation in our atlases, which indeed appeared again in our updated analysis. However, on closer inspection, the large “single-color blobs” in Figure 1C do not reflect species-specific cell clusters. Rather, they result from limitations of the t-SNE dimensional reduction used for visualization. For example, although the large red area middle-left in Figure 1C might appear to be a *D. melanogaster*-specific cluster, the cells within this area primarily map to our unannotated cluster 0—which actually also includes many *D. simulans* and *D. sechellia* cells (Reviewer Figure 3). These cells overlap more clearly when plotted in UMAP, underscoring the influence of the visualization method.

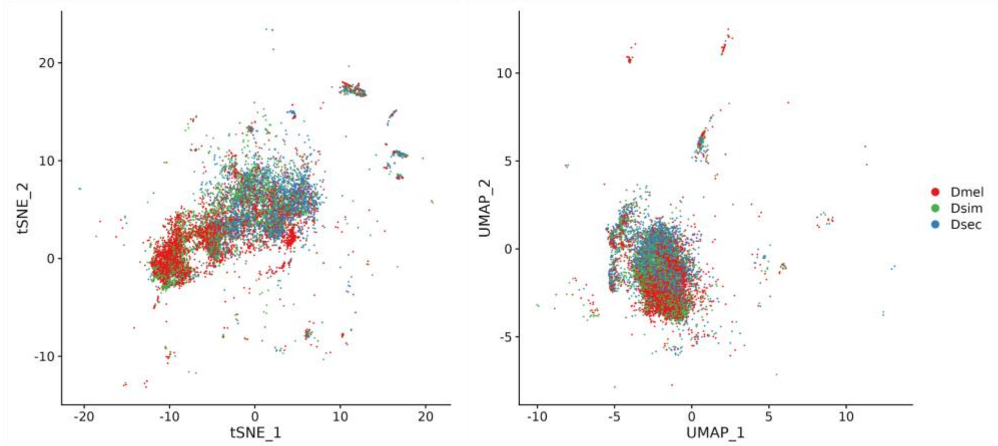

Reviewer Figure 3 | tSNE (left) and UMAP (right) plots of the unannotated cluster 0 from the integrated dataset.

While such clusters are not obviously species-specific, we appreciate the suggestion to investigate the large unannotated clusters more thoroughly. In this revision, we

have made several improvements to our analysis pipeline, including mapping each species' reads to their respective genomes, as described above. In response to this reviewer's comments we introduced iterative subclustering, as detailed in the Methods and summarized in Supplementary Figure 2. This approach allowed us to further dissect the large, previously unannotated clusters through a sequence of iterative subclustering, grouping, de-integration, and re-clustering steps, and by validating cell type calls through comparing integrated versus non-integrated atlases. Consequently, we were able to identify a total of 107 cell types - nearly twice as many as in our previous analysis - and now include 64% of all cells within annotated populations. Understanding the precise identity and functions of both annotated and unannotated cells remains an important challenge for the field; as this effort progresses in *D. melanogaster*, it might be possible to characterize other species-specific differences across our atlases in the future.

Other comments:
1. It is not justified or discussed why only female flies are used.

RESPONSE: Our primary goal of using only females was to focus on species-specific differences and minimize variation from other biological factors, including sex, which might affect cell type composition and gene expression. The choice of sex was arbitrary, but in part guided by our focus on females in many of our prior studies (e.g., noni attraction (Auer, Nature 2020; Takagi Nature Comm 2024) and oviposition (Álvarez-Ocaña et al. Nature Comm 2023)). Of course, analysis of male flies might reveal other species-specific differences, but this is beyond the budget/scope of the current project. We addressed this point in the Discussion section as follows:

"The choice of sex was arbitrary, but in part guided by our focus on females in many of our prior investigations (e.g., noni attraction [33,42] and oviposition [34]). In the future, comparative studies on males will reveal sex-specific or sex-neutral species differences in drosophilid central brains."

2. When comparing DEGs across cell types, does the cell number affect DEG numbers?

RESPONSE: We examined the correlation between cell number and the number of DEGs and observed that cell types with very few cells tend to yield fewer DEGs, likely due to reduced statistical power. However, for larger cell populations, we did not observe a comparable correlation. As illustrated in the new Supplementary Figure 10, the higher DEG counts observed in glial cell types are not driven solely by their relatively large cell numbers.

3. In the part of "Gene expression plasticity in the specialist brain", it is good to do and show the difference among the groups in Fig. 6a with statistical testing.

RESPONSE: We thank the reviewer for the suggestion. In response, we have now incorporated statistical testing among the groups in Figure 6A and have updated both the figure and its legend.
